# Angiopoietin/Tie2 signalling and its role in retinal and choroidal vascular diseases: a review of preclinical data

**DOI:** 10.1038/s41433-020-01377-x

**Published:** 2021-02-09

**Authors:** Antonia M. Joussen, Federico Ricci, Liliana P. Paris, Claudia Korn, Carlos Quezada-Ruiz, Marco Zarbin

**Affiliations:** 1 Department of Ophthalmology, Charité—Universitätsmedizin Berlin, Berlin, Germany; 2 Department of Experimental Medicine, Tor Vergata University of Rome, Rome, Italy; 3 F. Hoffmann-La Roche Ltd., Basel, Switzerland; 4 Genentech, Inc., South San Francisco, CA USA; 5 Retina y Vitreo, Clinica de Ojos Garza Viejo, San Pedro Garza Garcia, Mexico; 6 Institute of Ophthalmology and Visual Science, Rutgers New Jersey Medical School, Newark, NJ USA

**Keywords:** Mechanisms of disease, Medical research

## Abstract

The **ang**opoietin/**t**yrosine kinase with **i**mmunoglobulin and **e**pidermal growth factor homology domains (Ang/Tie) pathway is an emerging key regulator in vascular development and maintenance. Its relevance to clinicians and basic scientists as a potential therapeutic target in retinal and choroidal vascular diseases is highlighted by recent preclinical and clinical evidence. The Ang/Tie pathway plays an important role in the regulation of vascular stability, in angiogenesis under physiological and pathological conditions, as well as in inflammation. Under physiological conditions, angiopoietin-1 (Ang-1) binds to and phosphorylates the Tie2 receptor, leading to downstream signalling that promotes cell survival and vascular stability. Angiopoietin-2 (Ang-2) is upregulated under pathological conditions and acts as a context-dependent agonist/antagonist of the Ang-1/Tie2 axis, causing vascular destabilisation and sensitising blood vessels to the effects of vascular endothelial growth factor-A (VEGF-A). Ang-2 and VEGF-A synergistically drive vascular leakage, neovascularisation and inflammation, key components of retinal vascular diseases. Preclinical evidence suggests that modulating the Ang/Tie pathway restores vascular stabilisation and reduces inflammation. This review discusses how targeting the Ang/Tie pathway or applying Ang-2/VEGF-A combination therapy may be a valuable therapeutic strategy for restoring vascular stability and reducing inflammation in the treatment of retinal and choroidal vascular diseases.

## 摘要

本篇文章是关于不同照明条件对原发性开角型青光眼 (POAG) 患者视力和生活质量影响的研究的系统回顾。研究对CINAHL, MEDLINE, PsycARTICLES, PsycINFO, Embase and Ovid Nursing Database六个数据库进行系统文献检索, 截止发表日期为2019年4月。检索内容包括诊断为POAG的患者, 在变换照明设备/光照水平或炫光的情况下评估人群的视功能和生活质量。两名研究者独立筛选符合标准的受试者。从实验设计, 入选者标准, 结果与结论中挑选合格的研究并提取数据。入选研究的质量经过了严格的评估。

在8437项研究中, 共有56项研究符合入选标准。在POAG患者中调查光照对以下因素的影响: 生活质量 (18/56),心理物理学干预 (16/56), 功能性视力 (10/56), 日常活动 (10/56) 和定性发现 (2/56)。

POAG会影响患者的低亮度对比敏感度, 炫光症状, 暗适应的时间和程度。在视觉生活质量调查问卷中, 根据POAG患者反馈, 照明设备, 炫光和暗适应的问题较其它问题更多见。这些问题随着进行性视野的缺失而严重, 与同年龄对照组相比(AMC), POAG患者在发病的早期在低亮度和不同亮度切换的环境中会面临更多的困难, 这对之前POAG患者早期阶段没有症状的认知进行了挑战。但是, 基于性能方面的研究很少显示POAG参与者和AMC在模拟非最佳照明条件下日常活动方面有显着差异。 需要对较大的样本进行进一步研究, 以优化环境照明和面向任务的照明, 以支持患者适应POAG。未来亟待大样本的研究为POAG患者提供优化环境和适合工作的照明。

## Introduction

The **ang**iopoietin/**t**yrosine kinase with **i**mmunoglobulin and **e**pidermal growth factor homology domains (Ang/Tie) pathway plays an important role in the maintenance of vascular stability. The ligand angiopoietin-1 (Ang-1) binds to and phosphorylates the Tie2 receptor, thus activating it and promoting cell survival and vascular stability. Angiopoietin-2 (Ang-2), upregulated under pathological conditions, acts as a context-dependent agonist/antagonist of the constitutive Ang-1/Tie2 axis, promoting development of pathological features. As such, evidence from preclinical and early-phase clinical studies suggests that the Ang/Tie pathway regulates vascular stability and the inflammatory response, which could be valuable to the treatment of retinal and choroidal diseases [1].

Retinal and choroidal vascular diseases, such as diabetic retinopathy (DR)/diabetic macular oedema (DMO), neovascular age-related macular degeneration (nAMD) and retinal vein occlusion (RVO), are leading causes of blindness and visual impairment worldwide. A common feature of these conditions is destabilisation of the mature vasculature, which is associated with increased vascular permeability, inflammation and/or growth of pathological new vessels. Treatment of retinal and choroidal vascular diseases was revolutionised in the first decade of the 21st century with the introduction of vascular endothelial growth factor (VEGF)-blocking agents, which have markedly improved outcomes for patients with these conditions [2–4]. However, some patients do not fully respond to anti-VEGF monotherapy, and frequent injections are required to maintain visual gains, highlighting the multi-factorial nature of these diseases [3, 5, 6]. A deeper understanding of retinal biology will enable one to identify additional pathophysiological pathways, understand the basis of limitations of current therapy and develop novel multi-targeted therapeutic approaches for more effective treatment of retinal and choroidal diseases.

This article reviews preclinical data on the Ang/Tie pathway and describes its relevance both in physiological and pathophysiological conditions, focussing on retinal vascular and choroidal diseases.

## The Ang/Tie signalling pathway in angiogenesis

**Fig. 1:**
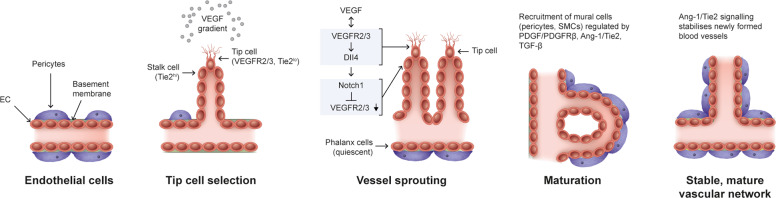
Angiogenic cascade. Steps in the angiogenic cascade: formation of a stable mature vascular network requires vessel sprouting, maturation and vessel remodelling. The collective migration of ECs is led by a Tie2^lo^ tip cell that guides the Tie2^hi^ stalk cells to elongate a vessel in response to a VEGF gradient. VEGF/VEGFR2/3 signalling in tip cells induces Dll4 expression in these cells, which then signals via Notch1 in stalk cells to downregulate VEGFR2/3, thereby inhibiting tip cell fate in stalk cells. Migrating tip cells anastomose with tip cells from neighbouring sprouts, while the trailing stalk cells proliferate to elongate the sprout and form a vascular lumen. Following perfusion of these vessels, ECs gain stability and form a monolayer of quiescent phalanx cells connected by vascular endothelial cadherin and claudins. Formation of a basement membrane and recruitment of mural cells (SMCs and pericytes) occurs in a process regulated by PDGF/PDGFRβ, Ang-1/Tie2 and TGF-β signalling, stabilising the vasculature. Cells are not represented to scale. Ang-1 angiopoietin-1, Dll4 delta-like 4, EC endothelial cell, PDGF platelet-derived growth factor, PDGFRβ platelet-derived growth factor receptor-β, SMC smooth muscle cell, Tie2^hi^ tyrosine kinase with immunoglobulin and endothelial growth factor homology domains 2 high, Tie2^lo^ tyrosine kinase with immunoglobulin and endothelial growth factor homology domains 2 low, TGF-β transforming growth factor-β, VEGF vascular endothelial growth factor, VEGFR vascular endothelial growth factor receptor.

Angiogenesis is a multi-step process involving vessel sprouting, vessel maturation and vessel remodelling [7, 8]. Vessel sprouting is mediated by collective migration of endothelial cells (ECs) led by a ‘tip cell’ that guides ‘stalk cells’ to elongate a vessel in the presence of factors, including VEGF receptors and Notch ligands [7, 9, 10] (Fig. 1). Signalling through VEGF receptors allows tip cells to guide stalk cells along VEGF gradients [7]. As tip cells anastomose with cells from surrounding sprouts, stalk cells elongate and form a lumen, and proliferate to form new vessels and branches [8]. Once these newly formed vessels are perfused, vessel maturation occurs as ECs gain stability and form a monolayer of quiescent phalanx cells connected by vascular endothelial cadherin and claudins [7, 11]. Formation of a basement membrane and recruitment of mural cells (vascular smooth muscle cells and pericytes), a process regulated by platelet-derived growth factor (PDGF)/PDGF receptor-β, Ang-1/Tie2 and transforming growth factor-β (TGF-β) signalling further stabilises the vasculature (Fig. 1) [7, 8]. Subsequent vascular remodelling involves regression of redundant branches to adapt to the metabolic demands of the tissue [12].

The Ang/Tie pathway, a key player in the multi-step angiogenic cascade, consists of two type I tyrosine kinase receptors (Tie1, Tie2), and four ligands (Ang-1, Ang-2, Ang-3, Ang-4) [13]. While Ang-1 and Ang-2 have been studied in depth, Ang-3 and Ang-4 are less well characterised [1]. The receptor components of the Ang/Tie pathway, Tie1 and Tie2, are expressed primarily in the endothelium, although they have also been detected on haematopoietic cells [14], and in the case of Tie2, also on pericytes [15]. Tie1 classically has been described as an orphan receptor that modulates surface presentation and activation of Tie2 by Ang-1 and Ang-2 [16]. In addition, a family of eight angiopoietin-like ligands has also been identified. Although structurally similar to angiopoietins, the angiopoietin-like ligands do not bind to either of the Tie receptors, but signal through leucocyte immunoglobulin-like receptors (LILRs) and contribute to the regulation of angiogenesis, inflammation and metabolism [17]. Independently of Tie2, Ang-2 can also bind to integrins to promote vascular destabilisation [18, 19].

Ang-1 is a Tie2 receptor agonist and is expressed by mesenchymal cells, pericytes and smooth muscle cells [1, 13]. In healthy vessels and resting ECs, Ang-1/Tie2 signalling promotes vascular stabilisation [1, 13]. Pericyte-derived Ang-1 binds to and induces phosphorylation and activation of Tie2 on ECs, leading to clustering of Ang-1/Tie2 complexes at cell–cell junctions, downstream activation of the phosphatidylinositol 3-kinase/protein kinase B (PI3K/AKT) pathway and induction of downstream survival effectors, including endothelial nitric oxide synthase and survivin [1, 20–22]. Additionally, Tie2 activation phosphorylates transcription factor forkhead box protein O1 (FOXO1) and prevents its nuclear translocation, consequently inhibiting transcription of its target genes. One of these genes is Ang-2, which competes with Ang-1 for Tie2 and induces EC destabilisation [1, 23]. Ang-1/Tie2–mediated signalling also activates A20-binding inhibitor of nuclear factor kappa-light-chain-enhancer of activated B cells (ABIN), which inhibits nuclear factor kappa B (NFκB) activation [24], and thus expression of inflammatory genes, such as intracellular cell adhesion molecule-1 (ICAM-1), vascular cell adhesion molecule-1 (VCAM-1) and E-selectin [25]. Ang-1/Tie signalling via GTPase pathways (Rac1/Rap1 or IQ domain GTPase-activating protein 1 [IQGAP]/Rap1) results in cortical actin cytoskeleton stabilisation and improves endothelial integrity [26]. Ang-1/Tie2 signalling and its downstream effects therefore promote EC survival and suppress further proliferation, stabilising newly formed blood vessels and forming a more efficient and stable vascular network [1, 13]. In migrating ECs, Tie2 is localised at the cell–extracellular matrix interface and preferentially activates extracellular signal–regulated kinase signalling [22, 27]. Recruitment of adaptor proteins, such as downstream of tyrosine kinase–related protein and growth factor receptor–bound protein 14, to the Tie2 receptor supports PI3K–mediated EC migration [1, 13]. Overall, the Ang-1/Tie2 signalling axis promotes vascular stabilisation and quiescence under physiological conditions.

**Fig. 2:**
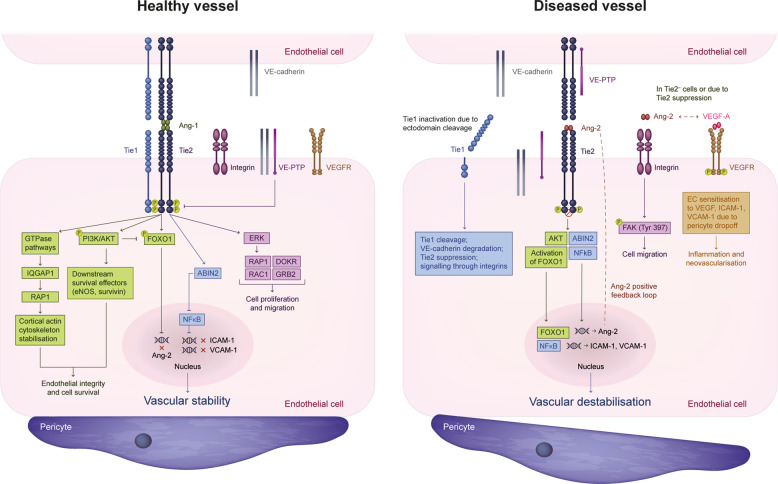
Ang/Tie signalling pathway under physiological and pathophysiological conditions. The Ang/Tie pathway regulates vascular stability under physiological and pathological conditions. The receptor components of the Ang/Tie pathway, Tie1 and Tie2, are expressed primarily in the endothelium. In a healthy vessel (left), Ang-1/Tie2 signalling at cell–cell junctions leads to downstream activation of the PI3K/AKT pathway and induction of eNOS and survivin, leading to EC survival. Tie2-mediated phosphorylation of FOXO1 prevents its nuclear translocation, inhibiting transcription of its target genes, including Ang-2, while inhibition of NFκB cells suppresses the expression of inflammatory genes such as ICAM-1, VCAM-1 and E-selectin. Ang-1/Tie2 signalling via GTPase pathways (Rac1/Rap1 or Iqgap1/Rap1) results in cortical actin cytoskeleton stabilisation. In migrating ECs, Tie2 is localised at the cell–extracellular matrix contacts and preferentially activates ERK signalling. Recruitment of adaptor proteins such as DOKR and GRB2 to the Tie2 receptor supports PI3K-mediated EC migration. Overall, Ang-1/Tie2 signalling and its downstream effects promote EC integrity, contributing to vascular stability. In a diseased vessel (right), Ang-2/Tie2 signalling leads to pericyte detachment, which sensitises the retinal vasculature to VEGF and other proinflammatory factors via activation of FOXO1 target genes, downregulation of Tie1 and consequent suppression of Tie2. Cells are not represented to scale. ABIN2 A20-binding inhibitor of nuclear factor kappa B, AKT protein kinase B, Ang angiopoietin, Ang-1 angiopoietin-1, Ang-2 angiopoietin-2, DOKR Dok-related protein, EC endothelial cell, eNOS endothelial nitric oxide synthase, ERK extracellular signal–regulated kinase, FAK focal adhesion kinase, FOXO1 forkhead box protein O1, GRB2 growth factor receptor–bound protein 2, ICAM-1 intracellular cell adhesion molecule-1, Iqgap1 IQ domain GTPase-activating protein 1, NFκB nuclear factor kappa B, P phosphorylated, PI3K phosphatidylinositol 3-kinase, Tie tyrosine kinase with immunoglobulin and endothelial growth factor homology domains, Tyr tyrosine, VCAM-1 vascular cell adhesion molecule-1, VE-cadherin vascular endothelial cadherin, VEGF vascular endothelial growth factor, VEGF-A vascular endothelial growth factor-A, VEGFR vascular endothelial growth factor receptor, VE-PTP, vascular endothelial protein tyrosine phosphatase.

Ang-2 is produced mainly by ECs and stored in Weibel–Palade bodies. Ang-2/Tie2 signalling in ECs leads to pericyte detachment, which sensitises the retinal vasculature to VEGF and other proinflammatory factors via activation of FOXO1 target genes (including Ang-2, creating a positive feedback loop), downregulation of Tie1, and consequent suppression of Tie2 [1, 28]. A Tie2-low environment induces Tie2-independent Ang-2 signalling through integrins on ECs [18]. Ang-2 signalling via β1-integrin in Tie2-silenced ECs or in Ang-2 transgenic mice promotes changes in the actin cytoskeleton, affecting vascular endothelial cadherin-mediated EC–EC adhesion and cell–extracellular matrix adhesion, resulting in vascular destabilisation [19]. Effects of Ang-2 signalling on ECs are context dependent and modulated by several factors [16]. First, EC type (tip, stalk), which determines expression levels of Tie2. In angiogenic tip cells, which express low levels of Tie2, Ang-2 signals independently of Tie2 by binding directly to integrins, promoting endothelial tip cell migration and vessel sprouting via focal adhesion kinase phosphorylation (Tyrosine 397) [18]. In stalk cells, Ang-2 expression is low while Tie2 expression is high, which favours Ang-1/Tie2 signalling, leading to vascular remodelling and stabilisation [18]. Second, presence of Tie1 might downregulate Tie2 surface expression in tip cells and sustain Tie2 signalling in stalk cells [29]. Under inflammatory conditions, Tie1 is inactivated due to ectodomain cleavage, which may convert Ang-2 from a Tie2 agonist to antagonist [1, 16]. Third, the ratio of Ang-1/Ang-2; in the absence of Ang-1, Ang-2 might act as a weak agonist of Tie2 [30]. Restoring Ang-1 signalling through Tie2 may therefore reverse the effects of Ang-2/Tie2 signalling and contribute to the stabilisation of vasculature. Finally, presence of modulators such as vascular endothelial protein tyrosine phosphatase (VE-PTP); VE-PTP is absent in the lymphatic endothelium, conferring agonist properties to Ang-2. Conversely, in vascular ECs, Ang-2 acts as a competitive antagonist due to presence of VE-PTP [31] (Fig. 2).

Ang-2 produced by ECs has been suggested to activate Tie2 receptors on pericytes in a paracrine fashion, causing pericyte detachment, in contrast to Ang-1, which activates pericyte Tie2, stabilising their association with ECs [15].

### Retinal and choroidal vasculature under physiological conditions

**Fig. 3:**
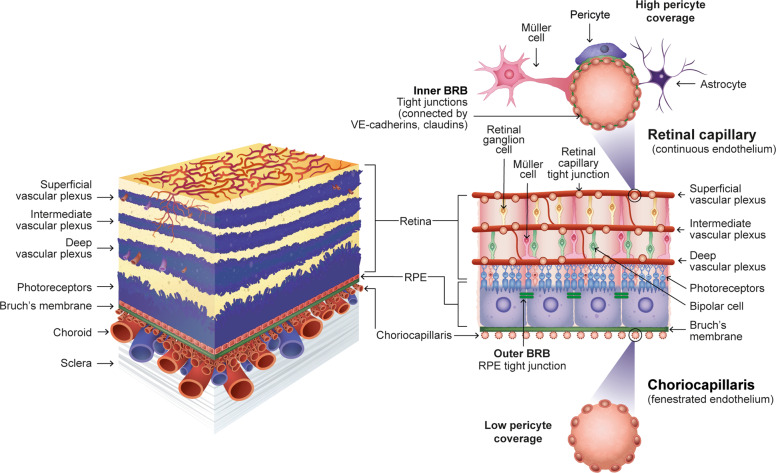
Retinal and choroidal vasculatures, inner and outer BRB. The retinal vasculature supplies the inner two-thirds of the retina, and the exchange of nutrients with the retinal tissue is highly regulated by the inner BRB, formed by tight junctions connecting retinal capillary ECs. The inner BRB is covered by astrocytes, Müller cells and a high density of pericytes. The choroidal vasculature supplies the outer one-third of the retina. The choriocapillaris is fenestrated and has low pericyte coverage. Tight junctions connecting the RPE cells form the outer BRB. Cells are not represented to scale. BRB blood–retinal barrier, EC endothelial cell, RPE retinal pigment epithelium, VE-cadherin vascular endothelial cadherin.

The retina is one of the most metabolically active tissues in the body. The layered neuronal architecture of the retina requires an extensive and stable vascular supply, which is provided by the retinal and choroidal vasculatures. The retinal vasculature supplies the inner two-thirds of the retina, and exchange of nutrients with retinal tissue is highly regulated by the inner blood–retinal barrier (BRB), formed by tight junctions connecting retinal capillary ECs [3]. The inner BRB is covered by astrocytes, Müller cells and a high density of pericytes (~95% coverage) [32], which, together with ECs, are organised in a neurovascular unit. In contrast, the choroidal vasculature supplies the outer one-third of the retina. The choriocapillaris is fenestrated, and pericyte coverage is low (~11% coverage). Tight junctions connecting retinal pigment epithelium cells form the outer BRB, ensuring a selective movement of solutes into the retina [3] (Fig. 3).

### Pathophysiological concepts in retinal and choroidal diseases

Hypoxia-induced vascular leakage and ischaemia, pathological neovascularisation and chronic inflammation are major causes of vision impairment in a wide range of retinal and choroidal diseases, such as DR, RVO and nAMD [3]. Hypoxia induces the translocation of hypoxia-inducible factor-1 and hypoxia-inducible factor-2 to the nucleus [33], where they enhance the transcription of VEGF and other proangiogenic factors, including PDGF-β, placental growth factor and stromal-derived factor [34]. Sustained low-grade inflammation in the retina and choroid is characterised by upregulation of inflammatory cytokines (e.g. interleukin-1β, interleukin-6, tumour necrosis factor-α [TNF-α]) and chemokines (e.g. monocyte chemoattractant protein-1, interleukin-8) produced by activated endothelial and microglial cells, resulting in leucocyte recruitment [35–37]. All these pathways lead to vascular destabilisation and vascular inflammation, with detrimental consequences for retinal function.

In nAMD, retinal pigment epithelium damage (associated with choriocapillaris dropout, hypoxia, oxidative stress and other cellular insults) may be associated with upregulation of VEGF and other proangiogenic factors that promote growth of immature and leaky neovessels, or choroidal neovascularisation (CNV) [38–41]. Given their leaky and fragile nature, these neovessels contribute to oedema, haemorrhaging and fibrosis, disrupting retinal function [42, 43]. Animal models to study nAMD pathology include the JR5558 mouse model, which develops spontaneous bilateral CNV [44], and laser-induced CNV mouse and cynomolgus monkey models [45–47].

In DR, chronic hyperglycaemia induces a proinflammatory state that promotes leukostasis, EC and pericyte apoptosis, BRB failure and retinal hypoxia [3, 48]. Pericyte dropout, a hallmark of DR, contributes to vascular destabilisation [28]. DMO, a vision-threatening manifestation of DR, occurs when leakage from the retinal vasculature causes fluid accumulation in the macula [3]. Vision loss in DR may occur due to development of retinal capillary nonperfusion, DMO (the most frequent cause) or neovascular complications associated with proliferative DR. DR pathophysiology has been studied mainly in rodents (e.g. in streptozotocin-induced diabetic mouse models [49, 50] and the *ob/ob* mouse model of type 2 diabetes [51]).

RVO is classified as either central RVO, hemicentral RVO or branch RVO. Initial obstruction to venous outflow leads to increased intravenous pressure, hypoxia in distribution of the area serviced by the occluded vessel, upregulation of proinflammatory cytokines (including VEGF, TNF-α and interleukin-1), inflammation and vascular leakage. Development of macular oedema is the primary cause of vision loss in RVO [3, 52]. As a later consequence, vascular remodelling or pathological neovascularisation (when extensive ischaemia is persistent) may develop. Branch and central RVO are most commonly studied in non-human primates, followed by rodents and pigs, all of which show retinal haemorrhages and ischaemia characteristic of RVO [53].

Despite anti-VEGF therapy becoming the standard of care for the treatment of nAMD and DR over the last 15 years and overall decreasing the incidence of blindness, there is still need for additional improvements, given that even in randomised clinical trials with intense monitoring and frequent regimens, only about ~44% of patients achieve minimum driving vision (best-corrected visual acuity [BCVA] of 20/40 Snellen equivalent) [54, 55], and over the long term, patients fail to sustain those initial BCVA gains achieved during the core studies [56, 57]. Furthermore, >60% of patients with nAMD and DMO showed persistent fluid and retinal thickening, respectively, even after 2 years of anti-VEGF therapy [58, 59]. In addition, real-world data suggest that in many cases, patients in clinical practice do not receive optimal anti-VEGF dosing frequency, resulting in lower BCVA gains for patients in the real-world setting [5, 6], highlighting the need for more durable agents that are able to provide sustained efficacy through extended durability both in clinical trials and in clinical practice. Furthermore, anti-VEGF monotherapy does not address inflammation and fibrosis, frequently associated with advanced stages of retinal and choroidal vascular diseases. Given the role of the Ang/Tie signalling pathway in the maintenance of vascular homoeostasis and the evidence of upregulation of Ang-2 in retinal and choroidal diseases [60], new treatments targeting this pathway may be beneficial in reducing vascular destabilisation associated with neovascularisation as well as inflammation.

### The Ang/Tie signalling pathway under pathological conditions and its therapeutic modulation

#### Leakage and neovascularisation

Intravitreal administration of Ang-1 was shown to reverse diabetes-induced damage to the retinal vasculature in rodent models by reducing EC injury and BRB breakdown, accompanied by reduced retinal endothelial nitric oxide synthase expression, nitric oxide levels, AKT and mitogen-activated protein kinase activity and ICAM-1 expression [61]. Furthermore, in a mouse model of nAMD, Tie2 activation via VE-PTP inhibition by its pharmacological inhibitor AKB-9778 suppressed neovascularisation and provided further evidence on modulation of the Ang/Tie pathway and its therapeutic potential for treatment of retinal and choroidal diseases [62].

Ang-2 overexpression under pathological conditions, such as hyperglycaemia, mediates integrin-mediated pericyte detachment and apoptosis [28, 50], which destabilises the retinal vasculature [63]. In a transgenic mouse model with experimentally induced DR, Ang-2 overexpression (in mOpsinhAng2 mice) increased the number of circulating pericytes in diabetic retinas by 2.3 fold versus non-diabetic retinas (non-diabetic wild-type: 51 ± 7 and diabetic mOpsinhAng2 mice: 118 ± 25 pericyte per mm^2^ of capillary area; *P* < 0.0001). This effect was not seen when Ang-2 was absent in loss of Ang-2 function mouse models (Ang2LacZ mice) [64]. In another induced DR mouse model, astrocyte apoptosis and consequent retinal vascular leakage occurred via Ang-2/integrin activation, which was prevented by targeting this pathway [49]. At the cellular level, elevated glucose levels decreased Ang-1–induced phosphorylation of Tie2 and downstream AKT activation in large-vessel human ECs, suggesting that hyperglycaemia inhibits the protective effect of Ang-1 and promotes vascular destabilisation [65].

In laser-induced CNV rodent models of nAMD, modulating the Ang/Tie pathway by increasing Ang-1 levels and inhibiting VE-PTP–stabilised retinal and choroidal vasculature [45, 62, 66, 67]. Activating Tie2 through an Ang-2–binding and Tie2-activating antibody was shown to decrease VEGF-induced CNV leakage, relieving hypoxia and promoting maintenance of the choriocapillaris, while also reducing retinal pigment epithelium damage and CNV growth [68]. Furthermore, the number of spontaneously occurring CNV lesions was significantly reduced in JR5558 mice treated with anti–VEGF-A/Ang-2 antibody versus anti–VEGF-A alone (*P* = 0.0428) and anti–Ang-2 alone or immunoglobulin G (IgG)-treated controls (*P* < 0.0001). Similarly, dual Ang-2/VEGF-A inhibition significantly reduced CNV-induced leakage versus anti–VEGF-A alone (*P* = 0.0037) and anti–Ang-2 alone or IgG-treated controls (*P* < 0.0001) [47]. Similar results were obtained in a laser-induced CNV cynomolgus monkey model [47].

Dual inhibition of Ang-2 and VEGF-A significantly reduced pathological neovascularisation and vessel density by >50% (*P* = 0.04) versus IgG controls, and enhanced pericyte coverage on blood vessels versus IgG controls and anti–Ang-2 or anti–VEGF-A monotherapy by approximately two-fold (*P* ≤ 0.04) in a human breast tumour-bearing mouse model. These data suggest that dual targeting of Ang-2 and VEGF-A promotes a more mature phenotype of abnormal neovessels [69].

**Fig. 4:**
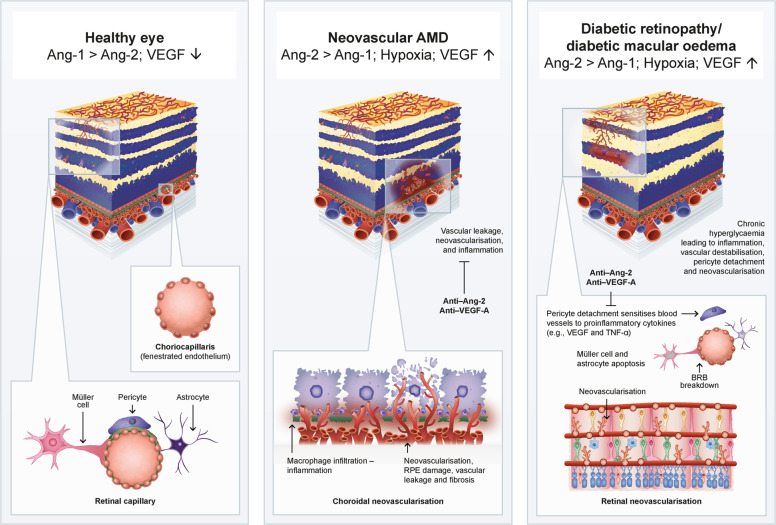
Overview of the effects of dual Ang-2/VEGF-A inhibition in nAMD and DR. Retinal and choroidal vasculatures in a healthy eye (left), an eye with nAMD (middle) and an eye with DR (right). Ang-2 and VEGF-A synergistically drive vascular leakage, inflammation and neovascularisation of choroidal vessels in nAMD, and neovascularisation and abnormal permeability of retinal vessels in DR. Data from preclinical studies suggest that combined blockade of Ang-2 and VEGF-A could act synergistically to reduce these effects, improving outcomes in retinal and choroidal diseases. Cells are not represented to scale. AMD age-related macular degeneration, Ang-1 angiopoietin-1, Ang-2 angiopoietin-2, BRB blood–retinal barrier, DR diabetic retinopathy, nAMD neovascular age-related macular degeneration, RPE retinal pigment epithelium, VEGF vascular endothelial growth factor, VEGF-A vascular endothelial growth factor-A.

Synergistic effects of Ang-2 and VEGF-A in driving vascular destabilisation, and data from preclinical studies, suggest that combination of Ang-2 blockade with anti-VEGF therapy could effectively reduce leakage, pathological neovascularisation and inflammation, thus potentially improving outcomes in retinal and choroidal diseases [70, 71] (Fig. 4).

#### Inflammation and neuroinflammation

The Ang/Tie pathway plays a key role in inflammation because it induces ICAM-1 and VCAM-1 expression through Ang-2/Tie2 signalling, promoting leucocyte adhesion and transmigration into inflamed tissues in response to inflammatory cytokines [72–74]. Previous studies in mouse tumour models of post-surgical adjuvant therapy demonstrated that an increase in Ang-2–mediated ICAM-1 expression contributed to the observed increase in CCL2-induced recruitment of tumour-promoting CCR2^+^Tie2^−^ macrophages [74]. Ang-2 was also shown to promote β2-integrin–dependent myeloid cell infiltration into various organs in a transgenic mouse model with inducible EC-specific Ang-2 expression [73]. In a mouse dorsal skinfold chamber model, TNF-α–induced leucocyte adhesion to the endothelium and subsequent tissue infiltration were reduced in response to thioglycolate in Ang-2 knockout mice versus controls due to reduced expression of EC adhesion molecules [72]. In a different study, Ang-2–deficient mice showed significantly reduced VEGF-induced tracheal leakage versus wild-type controls. A similar effect on vascular leakage was seen with other proinflammatory cytokines, such as histamine and bradykinin [75]. Furthermore, in mouse models of myocardial infarction [76] and multiple sclerosis [77], Ang-2 blockade decreased proinflammatory polarisation of myeloid cells via integrins.

In retinal and choroidal diseases, including DR [28, 50], RVO [78] and nAMD [68], Ang-2 signalling regulates pathological events, such as pericyte detachment, increased vascular permeability, neovascularisation and inflammation. In rodent models of diabetes, modulating the Ang/Tie pathway through Ang-1 overexpression suppressed leucocyte adhesion by significantly reducing retinal ICAM-1 and VEGF protein levels versus controls (both *P* < 0.001) [61]. In a mouse model of spontaneous CNV (JR5558 mice), combined neutralisation of VEGF-A and Ang-2 with low, mid and high doses (3, 5 and 10 mg/kg) of bispecific anti–Ang-2/anti–VEGF-A antibody significantly reduced the number of Iba1+ macrophages around CNV lesions versus IgG controls (*P* < 0.0134 [low], *P* < 0.0078 [mid], *P* < 0.0048 [high]). Treatment with anti–Ang-2 and anti–VEGF-A alone also showed reduction in the number of Iba1+ macrophages, although these differences were not statistically significant versus IgG controls [47, 71].

Further evidence on benefits of dual Ang-2/VEGF-A inhibition was obtained from a mouse model of endotoxin-induced uveitis, in which dual Ang-2/VEGF-A inhibition with a bispecific anti–Ang-2/anti–VEGF-A antibody attenuated inflammatory response by reducing leucocyte infiltration into the inner retina versus untreated controls (*P* < 0.00001) or anti–VEGF-A alone (*P* < 0.0086) [47].

#### Fibrosis

Fibrosis, defined as excessive deposition of extracellular matrix, occurs as a consequence of a chronic wound healing response to tissue injury [79]. Even though the mechanism of fibrosis varies in different organ systems, some signalling pathways (e.g. TGF-β, PDGF, TNF-α, interferon gamma and fibroblast growth factor) and macrophage heterogeneity appear to be common mechanisms in development of myocardial, corneal, renal and retinal fibrosis [80–83].

Involvement of Ang-1 and Ang-2 in the pathogenesis of fibrosis was studied in transgenic mice ectopically expressing Ang-1, Ang-2 or murine VEGF_164_ in various combinations in cardiac tissue [84]. Double-transgenic mice with increased co-expression of Ang-2 and VEGF-A showed aberrant angiogenesis and severe fibrosis. Conversely, inducing Ang-1 expression (triple-transgenic mice) improved the phenotype [84]. In another study, Ang-1–deficient mice showed a stronger angiofibrotic response, with faster wound closure of an ear punch versus wild-type controls, suggesting that Ang-1 is critical in controlling angiofibrotic wound healing response [85]. Ang-1 activation of Tie2 was also shown to be critical in controlling fibrotic response to diabetic kidney injury. Hyperglycaemia caused only mild kidney damage in wild-type diabetic mice; however, lethal glomerulosclerosis-induced severe kidney failure occurred in 20% of the diabetic Ang-1–deficient cohort [85].

Fibrosis is a cause of irreversible vision loss in nAMD [81]. The mechanistic understanding of subretinal fibrosis and a potential involvement of the Ang/Tie pathway remain poorly understood and warrant further research.

## Discussion and conclusions

The Ang/Tie pathway plays an important role in the maintenance of vascular stability, which is mediated by a balance between the agonistic effect of Ang-1 on Tie2, and the antagonist effect of Ang-2. Preclinical evidence suggests that modulation of the Ang/Tie pathway, along with VEGF-A inhibition, can restore vascular stability by enhancing pericyte coverage and BRB integrity, thus reducing vascular leakage, pathological neovascularisation and tissue infiltration by inflammatory cells. In addition, Ang-2 inhibition also reduces proinflammatory macrophage polarisation and vascular responsiveness to VEGF and other proinflammatory cytokines, potentially contributing to prevention of sustained retinal inflammation [47, 62, 72–76]. Furthermore, modulation of the Ang/Tie pathway may have anti-fibrotic effects [84, 85], although its relevance and implication in retinal and choroidal diseases require further research.

Combined Ang-2 and VEGF-A inhibition may permit the homeostatic benefits of a neovascular response to hypoxia while attenuating the destructive effects of uncontrolled vascular permeability characteristic of macular and retinal neovascularisation in nAMD and DR, respectively. Dual inhibition of Ang-2 and VEGF-A and its effect on stabilisation of blood vessel permeability induced by inflammation, hyperglycaemia and/or hypoxia may also help consolidate benefits of anti–VEGF-A therapy on macular oedema of various aetiologies, and thus promote extended durability. This reasoning suggests that dual Ang-2/VEGF-A targeting may be a more beneficial therapeutic approach versus anti-VEGF monotherapy [1, 47, 60, 69, 71].

Our increasing understanding of the Ang-2/Tie pathway has contributed to redefining the therapeutic goal in nAMD from destruction of CNV lesions to therapy aimed at vascular stabilisation through suppression of CNV leakage. Harnessing the benefits of the dual inhibition approach along with Tie2 activation by Ang-1 [68] may be a desirable approach to maintain the choriocapillaris and stabilise the pathologic vasculature in nAMD by suppressing CNV-induced leakage [47, 68]. Stable CNV could potentially act as a ‘surrogate choriocapillaris’ when the existing vasculature becomes compromised, and attenuate long-term complications [86–89].

Rather than complete elimination of CNV lesions, induction of maturation and normal physiological function of the new vessels and, in the case of DMO, stabilisation and remodelling of damaged, leaking vessels, may lead to superior clinical outcomes versus VEGF blockade alone. Combination therapy with Ang-2/VEGF-A blockade may afford this opportunity in treatment of retinal diseases by promoting perfusion of hypoxic tissue via new vessels that have features of mature (vs. immature) vasculature or via stabilisation of previously damaged vasculature (i.e. intact tight junctions with no leakage or development of inflammation, fibrosis and other complications in the long term).

Combination therapies to enhance anti-VEGF effects, such as pegleranib (dual PDGF and VEGF targeting) have been investigated previously [90]. Pegleranib aimed at rendering vessels more susceptible to anti-VEGF action by disrupting their pericyte coverage. However, this combination did not meet the primary endpoint of superiority over anti-VEGF monotherapy in phase 3 trials [90]. In contrast, dual inhibition of Ang-2 and VEGF-A aims at reinforcing/restoring vascular stability by enhancing pericyte coverage, EC–EC adhesion and reducing perivascular inflammation along with sensitivity to VEGF, via inhibition of Ang-2.

Clinical trials are ongoing to determine the effect of combined Ang-2/VEGF-A inhibition on vascular stabilisation. For retinal and choroidal diseases, three molecules targeting the Ang/Tie pathway have been studied in phase 2 trials for DMO and nAMD. These include the VE-PTP inhibitor AKB-9778 (Aerpio Pharmaceuticals, Inc.) [91], anti–Ang-2 antibody nesvacumab and aflibercept combination therapy (Regeneron Pharmaceuticals, Inc.) [92] and the bispecific anti–Ang-2/anti–VEGF antibody faricimab (F. Hoffmann-La Roche Ltd.) [93, 94]. Efficacy of AKB-9778 was assessed in patients with DMO and non-proliferative DR. Despite overall improvements in central subfield thickness (significant at month 3; *P* = 0.008) in patients with DMO [95] and a trend in improvement of Diabetic Retinopathy Severity Scale score in patients with non-proliferative DR [91], there were no significant vision gains [95]. The nesvacumab/aflibercept combination, which was evaluated for treatment of DMO and nAMD, demonstrated a safe profile and a positive trend toward anatomic and Diabetic Retinopathy Severity Scale score improvement, but there were no visual gains versus monotherapy [96, 97]. While AKB-9778 is being tested as a topical formulation due to encouraging results in intraocular pressure, the nesvacumab/aflibercept combination did not progress to a phase 3 trial [91, 92]. Faricimab, a bispecific antibody that independently binds and neutralises both Ang-2 and VEGF-A, has demonstrated potential for sustained efficacy in phase 2 trials and is currently undergoing phase 3 clinical trials in patients with DMO and nAMD [93, 98].

The key potential benefits of dual Ang-2/VEGF-A inhibition in retinal diseases raises several new questions: which clinical/imaging endpoints will effectively assess its effects on vascular stability, inflammation, neurovascular unit integrity and fibrosis early in the disease course? How much dual Ang-2/VEGF-A inhibition is required to achieve desired clinical outcomes? Measuring outcomes of dual Ang-2/VEGF-A targeting might require adaptation versus current state-of-the-art evaluation of anti–VEGF-A monotherapy. Moreover, identification and characterisation of responsive patient populations (e.g. those with chronic conditions) is an important point to evaluate. Development of more subtle assessment of disease outcomes, including inflammation and fibrosis, as well as BRB breakdown, might be facilitated through deep learning algorithms applied to high-resolution optical coherence tomography-A and optical coherence tomography-B data. Additionally, the role played by chronic inflammation and mechanisms underlying development of subretinal fibrosis warrant further research to gain a deeper understanding of DMO and nAMD pathophysiology.

Further research and clinical trial results will add to our understanding of potential disease-specific benefits of dual Ang-2/VEGF-A inhibition.

### Summary

#### What is known about this topic

- The Ang/Tie pathway plays an important role in the maintenance of vascular stability, which is mediated by a balance between the agonistic effect of Ang-1 on Tie2, and the antagonist effect of Ang-2.
- Preclinical evidence suggests that modulation of the Ang/Tie pathway, combined with VEGF-A inhibition, can restore vascular stability by enhancing pericyte coverage and BRB integrity, thus reducing vascular leakage, pathological neovascularisation, and tissue infiltration by inflammatory cells.
- In addition, Ang-2 inhibition also reduces proinflammatory macrophage polarisation and vascular responsiveness to VEGF and other proinflammatory cytokines, potentially contributing to prevention of sustained retinal inflammation. Modulation of the Ang/Tie pathway also may have anti-fibrotic effects.

#### What this study adds

- Combined Ang-2 and VEGF-A inhibition may permit the homoeostatic benefits of a neovascular response to hypoxia while attenuating the destructive effects of uncontrolled vascular permeability characteristic of macular and retinal neovascularisation in nAMD and DR, respectively.
- Dual inhibition of Ang-2 and VEGF-A and its effect on stabilisation of blood vessel permeability induced by inflammation, hyperglycaemia and/or hypoxia may also help consolidate benefits of anti-VEGF-A therapy on macular oedema of various aetiologies, and thus promote extended durability of therapy. Thus, dual Ang-2/VEGF-A targeting may be superior to anti-VEGF monotherapy.
- Modulation of the Ang-2/Tie pathway has contributed to redefining the therapeutic goal in nAMD from the destruction of CNV lesions to therapy aimed at vascular stabilisation through suppression of CNV leakage.
- Harnessing the benefits of the dual inhibition approach along with Tie2 activation by Ang-1 may enable maintenance of the choriocapillaris and stabilisation of the pathologic vasculature in nAMD by suppressing CNV-induced leakage.
- Stable CNV could potentially act as a ‘surrogate choriocapillaris’ when the existing vasculature becomes compromised, and attenuate long-term complications.
